# Factor XI: structure, function and therapeutic inhibition

**DOI:** 10.1007/s11239-024-02972-5

**Published:** 2024-04-16

**Authors:** Ahmed E. Ali, Richard C. Becker

**Affiliations:** 1 Department of Internal Medicine, Mayo Clinic, Rochester, MN USA; 2 Department of Internal Medicine, University of Cincinnati, Cincinnati, OH USA

**Keywords:** Factor XI Inhibitors, Research and development of new anticoagulants, Atrial fibrillation, Ischemic stroke

## Abstract

Arterial and venous thromboembolism is a major medical concern that requires therapeutic anticoagulation in various medical fields to prevent its drastic consequences. Despite significant advances in anticoagulant therapy, thrombosis remains a leading cause of morbidity and mortality worldwide. Traditional anticoagulants like heparin and vitamin K antagonists (VKAs) have shown efficacy in preventing and treating thrombosis but come with an inherent risk of bleeding due to their non-specific inhibition of multiple coagulation factors. Subsequent direct oral anticoagulants (DOACs), targeting specific factors such as Xa or thrombin, demonstrated improved safety profiles compared to VKAs, yet bleeding remains a concern. Accordingly, research is focused on developing anticoagulants with improved safety profiles. A safer class of anticoagulants would have broad appeal. The intrinsic pathway of coagulation, involving factor XI (FXI), has attracted attention as a potential target for safer anticoagulants. Preclinical studies and epidemiological data indicate that FXI deficiency or inhibition protects against thrombosis with minimal bleeding. Current research involves evaluating various FXI-directed strategies, and phase 2 studies have shown promising results in orthopedic surgery, atrial fibrillation, end-stage renal disease (ESRD), myocardial infarction, and ischemic stroke. Several agents, such as antisense oligonucleotides, monoclonal antibodies, small synthetic molecules, natural peptides, and aptamers, have been developed to inhibit FXI at different stages, offering potentially safer alternatives to traditional anticoagulants. However, the optimal balance between preventing thrombosis and the risk of bleeding associated with FXI inhibitors requires validation through extensive phase 3 clinical trials using definite clinical endpoints. Several of such trials are currently underway or planned to define the role of FXI inhibitors in clinical practice and determine the most suitable FXI inhibitor for each specific indication. The current review highlights the rationale behind developing FXI inhibitors, presenting the most advanced agents in development, summarizing completed clinical trials, and discussing ongoing research efforts.

## Structure

**Fig. 1:**
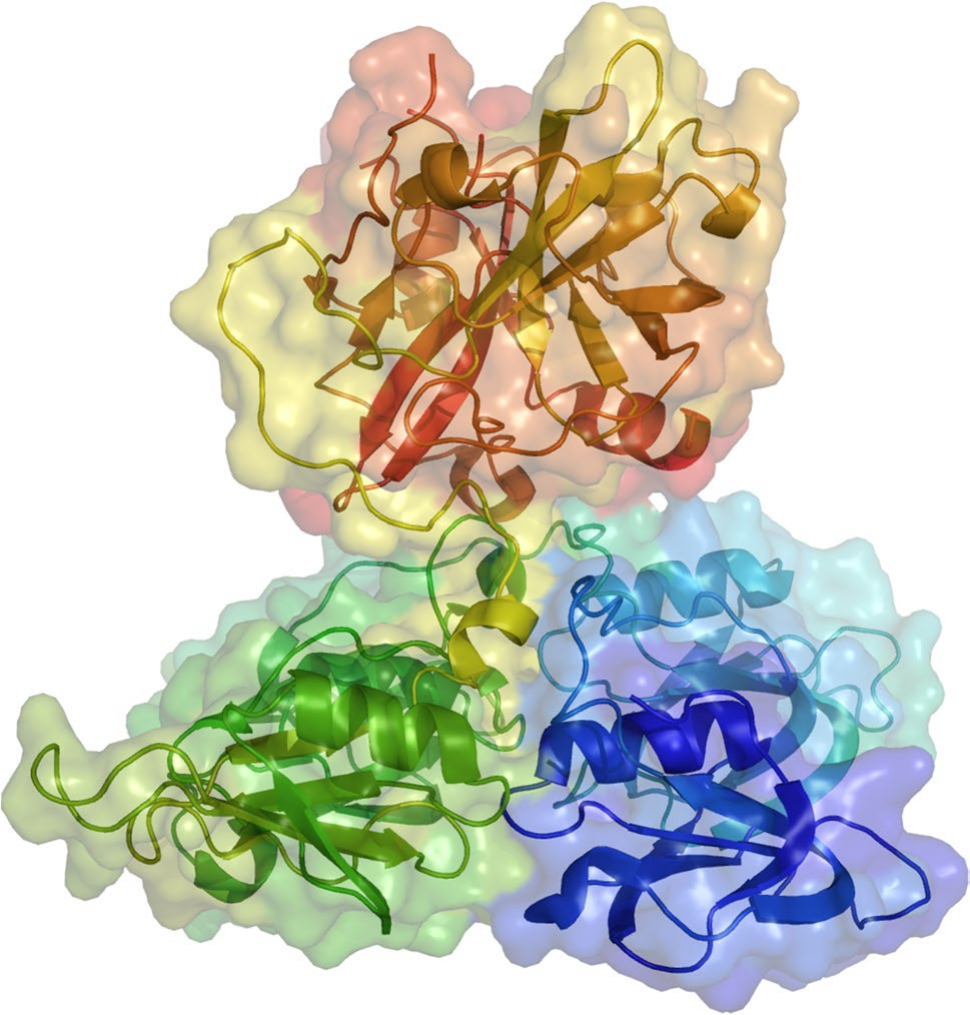
Factor XI Structure and Function. Based on crystallographic structure analysis, FXI contains 4 "apple domains" shown as ribbons in gold, magenta, green, and blue, that form a disk-like structure with extensive interfaces at the base of the catalytic domain. The characterization of the apple disk structure, and its relationship to the catalytic domain, provide important insights into the binding of factor XI with substrates like factor IXa, thrombin and its interaction with platelets through the glycoprotein (GP) Ib receptor

Factor XI (FXI), a 160-kDa glycoprotein serine protease, exists as a dimer with two identical subunits, each comprising 607 amino acids and linked by disulfide bonds. Each polypeptide is composed of a C-terminal light chain of 35 kDa, housing the trypsin-like catalytic domain, and an N-terminal heavy chain of 45 kDa, which contains four ~ 90 amino acid tandem repeats known as apple domains [1]. These apple domains play a crucial role in binding to various proteins; A1 interacts with high molecular weight kininogen (HK) and thrombin [2], A2 and A3 bind to FIX [3], A3 interacts with the platelet receptor GP1bα and heparin [4], and A4 binds to FXII and the other subunit of FXI [5, 6]. Activation of FXI occurs through cleavage by either XII or thrombin at the same site, leading to an intermediate structure termed 1/2FXIa, featuring one activated subunit [7]. Subsequently, FXIa activates FIX to FIXαβ by cleaving two activation sites [7]. While dimerization appears to be crucial for FXI activation, it is not a requirement for FIX cleavage, as monomeric FXI mutants can activate FIX at a similar rate as dimeric FXIa, albeit with reduced activation by FXII or thrombin [8] (Fig. 1).

## Rationale behind targeting factor XI for anticoagulation therapy

### Role of FXI in hemostasis

**Fig. 2:**
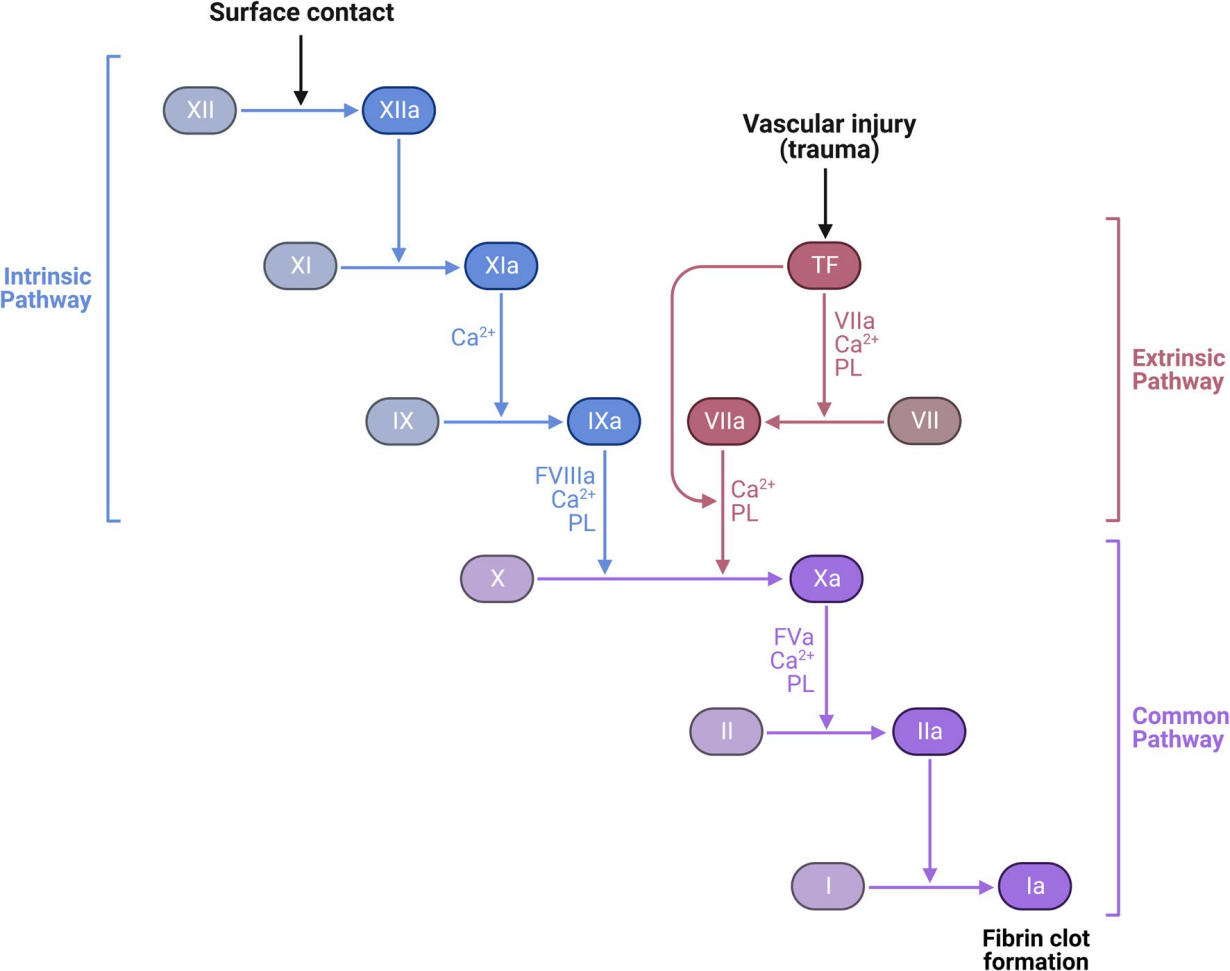
Intrinsic Coagulation Pathway. The intrinsic system of coagulation, also referred to as the intrinsic pathway includes surface contact activating factors XII and XI. Activated factor XI (XIa) in the presence of calcium enzymatically converts factor IX to its activated form, IXa. When combined with Factor VIIIa, calcium, and phospholipid from vascular/cellular surfaces the tenase complex converts prothrombin to thrombin that in turn converts fibrinogen to fibrin. Fibrin is the primary component of hemostatic and pathological thrombosis. The extrinsic pathway of coagulation follows vascular injury and exposure of tissue factor (TF) that convert FVII to VIIa, converting FX to FXa (common pathway of coagulation), and generating thrombin for fibrin clot formation

Hemostasis is initiated when tissue factor interacts with activated factor VII (TF-FVIIa) in what is called the tissue factor pathway. This interaction leads to the conversion of factor X into its active form, activated factor X (FXa). FXa then initiates a series of events that ultimately lead to the activation of factor II, leading to the formation of fibrin and the production of a hemostatic clot [9]. In fact, FXI has a relatively minor role in hemostasis where it is primarily activated to FXIa by thrombin, and this process consolidates the final hemostatic clot through activation of FIX [9]. The classic pathways of blood coagulation including intrinsic, extrinsic, common pathways and the role of FXI are illustrated in Fig. 2.

### Role of FXI in thrombosis

Unlike its more modest role in hemostasis, FXI plays a major role in pathological thrombus formation [10]. Pathological thrombosis can be triggered by two main pathways: the tissue factor pathway and the contact pathway. In either case, thrombin is generated during the initial phase, which subsequently activates Factor XI. In turn, FXI drives thrombus growth and propagation in a process called amplification [11]. This fundamental role of FXI in thrombosis is supported by several animal studies in which FXI-deficient or inhibited animal models had reduced incidence of thrombosis [12]. Furthermore, individuals with high levels of FXI were found to have a higher risk for venous thromboembolism (VTE) [13] while those with FXI deficiency had a lower incidence of DVT when compared to the general population [14].

Based on the different contributions of FXI in hemostasis and thrombosis discussed above, inhibition of FXI has emerged as a promising approach to uncouple the therapeutic benefits and the adverse effects of anticoagulant treatments; in other terms, by targeting FXI, it is possible that we might be able to reduce the risk of thrombotic complications without simultaneously increasing the likelihood of bleeding, which is a major concern with other types of anticoagulants.

## General properties

**Fig. 3:**
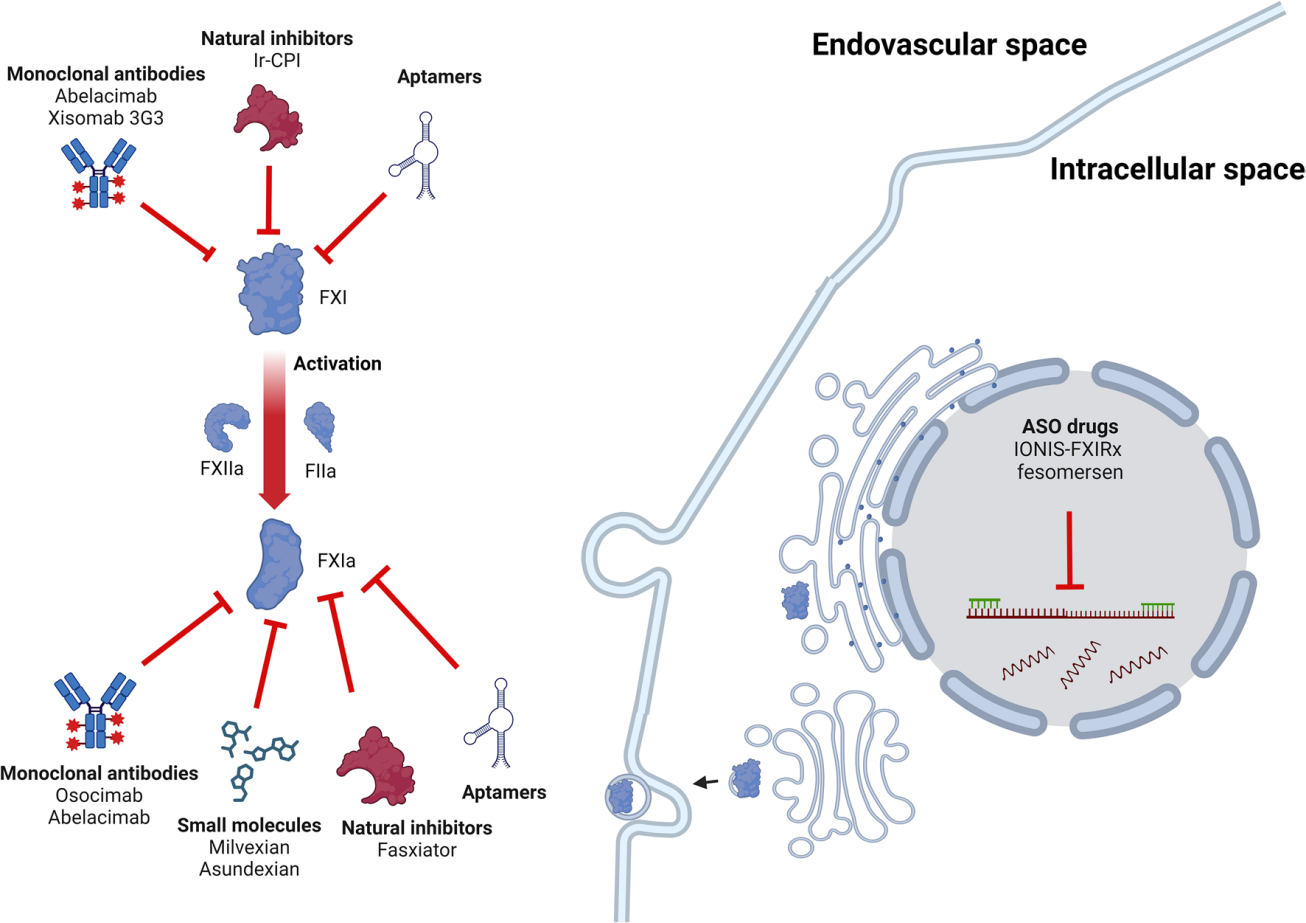
Sites of Factor XI Inhibition for Drugs in Development. Some inhibitors target factor XI while others bind factor XI in its activated form (XIa). Anti-sense oligonucleotides (ASO) inhibit the hepatic synthesis of factor XI in the intracellular space. Factor (F), Ixodes ricinus contact-phase inhibitor (Ir-CPI). The classes of inhibitors include monoclonal antibodies, small molecules, aptamers, anti-sense oligonucleotides, and natural inhibitors

**Fig. 4:**
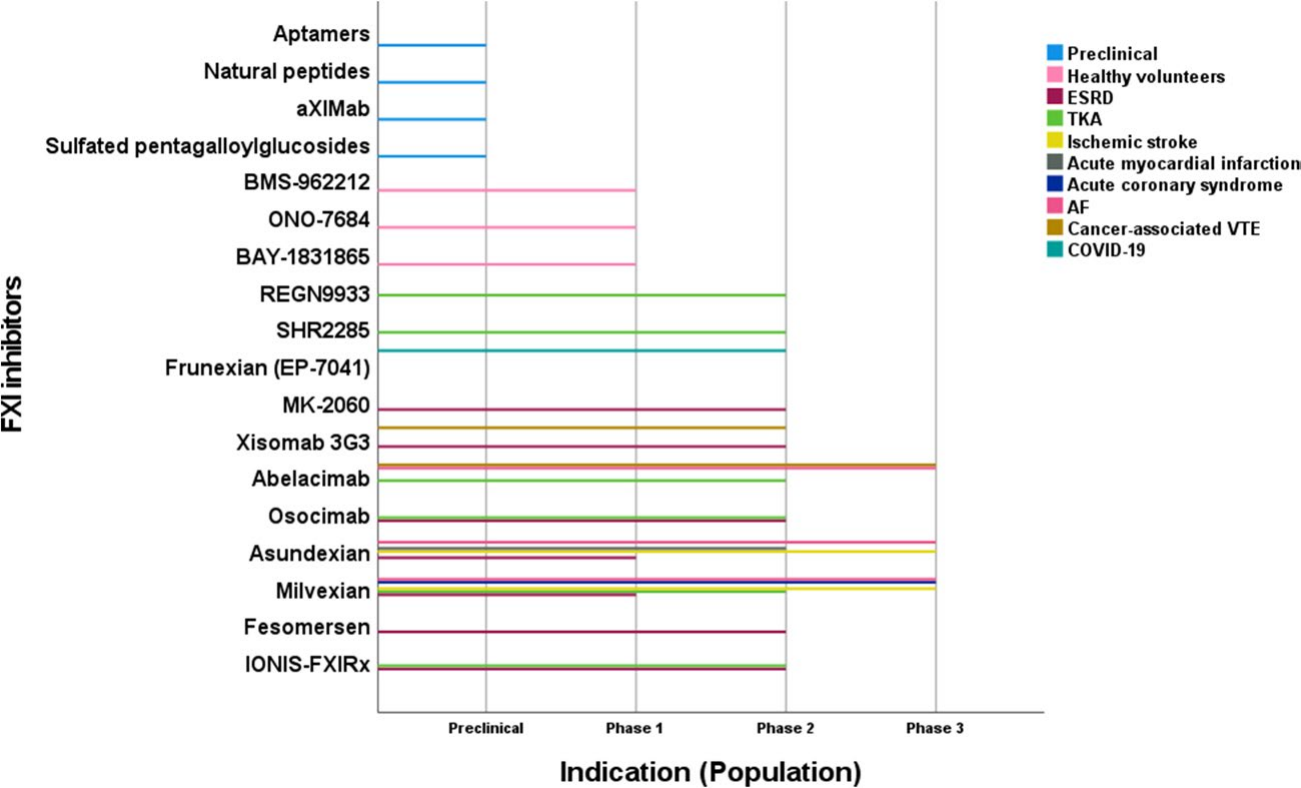
Representation of factor XI and factor XIa inhibitors in varied clinical conditions according to the phase of development in color coding format. ESRD (end-stage renal disease), AF (atrial fibrillation), TKA (total knee arthroplasty), VTE (venous thromboembolism)

Different inhibition strategies aimed at FXIa have been developed, and include antisense oligonucleotides, monoclonal antibodies, and small molecule inhibitors [15] (Fig. 3). Several of these inhibitors have demonstrated strong specificity for FXIa, leading to prolonged plasma clotting time, and have shown protective effects against thrombosis in different animal models without affecting the bleeding time [10, 16, 17]. The mechanisms, pharmacodynamics, and pharmacokinetics of FXI inhibitors that are tested in clinical trials are summarized in Table 1 [18–33]. FXI inhibitors in different stages of development are illustrated in (Fig. 4).

**Table 1:** Pharmacological and Pharmacokinetic characteristics of FXI inhibitors tested in clinical trials

	IONIS-FXIRx	Osocimab	Abelacimab	Xisomab 3G3	Milvexian	Asundexian
Class	ASO	Monoclonal antibody	Monoclonal antibody	Monoclonal antibody	Small molecule	Small molecule
Developer	Ionis Pharmaceuticals	Bayer HealthCare	Anthos Therapeutics, Novartis Pharmaceuticals	Aronora	Bristol-Myers Squibb, Janssen Pharmaceuticals	Bayer HealthCare
Tested doses on published clinical trials	200, 300 mg	Single intravenous postoperative doses of 0.3, 0.6, 1.2, 1.8 mg/kg; preoperative 0.3, 1.8 mg/kg	Single intravenous postoperative doses of 30, 75, 150 mg; monthly subcutaneous doses of 120, 180 mg	0.25, 0.5 mg/kg (phase 2); 0.1, 0.5, 2, 5 mg/kg (phase1)	25, 50, 100, 200 mg twice daily; 25, 50, 200 mg once Daily	20, 50 mg once daily (phase 2); 25, 50, 100 mg (phase 1)
Administration route	S.C	I.V or S.C	I.V or S.C	I.V	P.O	P.O
Tmax	Median of 6 h	1–4 h	1.75–2.00 h (after start of the 1 h infusion)	0.08–0.65 h	Median of 3 h	1–4 h
Terminal half-life	13.1–16.9 d	30–44 d	25–30 d	1.3–121 h	8.3–18.1 h	14–17.8 h
Effect of food	-	No	No	No	Slowed the rate of absorption, increased the rate of elimination, and increased the bioavailability	AUC decreased by 12.4%, C_max_ decreased by 31.4%. The median T_max_ increased from 2.50 to 5.00 h
Renal Elimination	No	No	No	No	Less than 20%	Less than 15%
Cyp P 450 metabolism	No	No	No	No	Yes, Cyp P450 (CYP)-3A4 (hepatic)	Minor role (no clinically relevant interaction)
Drug-drug interaction	No	No	No	No	Yes (Cyp P 450 inhibitors)	No
Indication in phase 2 trials	TKA, ESRD	TKA	TKA	ESRD	TKA, stroke	AF, stroke, AMI

*ASO* antisense oligonucleotides, *S.C* subcutaneous, *I.V* intravenous, *P.O* oral, *AUC* Area under the ROC Curve, *Cmax* peak plasma concentration, *Tmax* time to reach peak plasma concentration, *Cyp.P450* cytochrome P450, *TKA* total knee arthroplasty, *ESRD* end-stage renal disease, *AF* atrial fibrillation, *AMI* acute myocardial infarction.

### Antisense oligonucleotides

These drugs inhibit the synthesis of factor XI by binding to specific mRNA [34]. They are highly bound to plasma proteins with limited renal clearance. Additionally, they have a slow onset of action and long half-life. Therefore, and coupled with the 52-h half-life of FXI reversal of their action would require long-term FXI replacement with a factor XI concentrate [34]. IONIS-FXIRx was the first ASO drug that was tested in clinical trials [27, 35]. Another drug, fesomersen (LICA), a modified form of IONIS-FXIRx, is currently being studied [36].

### Small molecules

These drugs are synthetic compounds with low molecular weight. They inhibit FXIa through binding to target proteins; hence, their effects cannot be countered by replacement products, as they do not reduce factor XI levels directly. In certain situations, activated prothrombin complex concentrate, recombinant factor VIIa, and antifibrinolytic agents might be considered as potential interventions to counteract the action of these drugs. They have a rapid onset and short duration of action and are amenable to renal clearance. Milvexian [9, 18, 37, 38] and asundexian [20, 24, 30–32, 39] are two main drugs that have been widely tested in human clinical trials. Others include SHR-2285 [11], frunexian [40], ONO-7648 [41], BMS-962212 [42], and Sulfated pentagalloylglucosides [43].

### Monoclonal antibodies

This class of drug acts primarily by preventing activation of FXI to FXIa or by binding to FXIa and preventing its activity, however, there are other mechanisms as well. For example, Bay 1,831,865 hinders the binding of FXI to FIX, thereby preventing FXI activation. It also inhibits activation of FXI to FXIa [24]. Xisomab binds to FXI and to the A2 domain thereby preventing FXI activation by FXIIa [44]. The other functions of FXI are not impacted.

Monoclonal antibodies exhibit a rapid onset of action and a prolonged duration, with effects lasting over a month. Their metabolism relies mainly on the reticuloendothelial system [45]. In situations where their effects need to be reversed, the preferred method would be the use of tranexamic acid and agents that bypass FXI such as recombinant FVIIa (rFVIIa) or activated prothrombin complex concentrate (aPCC). Osocimab [46], Abelacimab [26], and Xisomab 3G3 [23] are three drugs that have been widely tested in human clinical trials. Other compounds that are under investigation or in the pre-clinical phase include BAY 1831865 [24, 47] and REGN9933 [48].

### Natural peptides

Fasxiator [49] and Ir-CPI [50] are natural peptides isolated from snake Bungarus fasciatus and tick Ixodes Ricinus respectively. They inhibit the activation of FXI with a rapid onset of action and a short half-life. Other natural peptides like Desmolaris, acaNAP10, and Boophilin are currently in the initial study phases on a limited basis [51–53]. Natural peptides inhibitors have not been tested in humans to date.

### Aptamers

DNA-based agents that inhibit either FXI or its activation to FXIa do so with considerable specificity, rapid onset of action and a short half-life [54]. They are in early stages of development and have not been tested in humans to date.

## Published clinical trials on factor XI inhibitors

Several phase 2 clinical trials investigating the safety and efficacy of FXI inhibitors have been published. These trials focused on six clinical indications for the use of FXI inhibitors which were total knee arthroplasty (TKA), end-stage renal disease (ESRD), atrial fibrillation (AF), acute non-cardioembolic ischemic stroke, acute ischemic stroke, or high-risk transient ischemic attack (TIA) and acute myocardial infarction. A summary of the published phase 2 clinical trials including indication, intervention, control, population, efficacy, and safety outcomes is shown in (Table 2).

**Table 2:** Summary of the published phase 2 clinical trials on Factor XI inhibitors

Indication	Trial name & Reference	Drug	Comparator	N	Primary efficacy and safety measures	Findings (Primary efficacy outcome)	Bleeding (safety outcome)
TKA	FXI-ASO TKA [56]	IONIS-FXIRx 200 and 300 mg S.C once daily	Enoxaparin 40 mg S.C once daily	300	Incidence of VTE. Incidence of major or CRNMB. (8–12 days after surgery)	IONIS-FXIRx 200 mg: 27% IONIS-FXIRx 300 mg: 4% Enoxaparin: 30% (The 200-mg regimen was noninferior, and the 300-mg regimen was superior, to enoxaparin)	IONIS-FXIRx 200 mg: 3% IONIS-FXIRx 300 mg: 3% Enoxaparin: 8%
	FOXTROT [25]	Osocimab 0.3, 0.6, 1.2, 1.8 mg/kg post-operative IV infusion and 0.3, 1.8 mg/kg pre-operative IV infusion	Enoxaparin 40 mg S.C once daily and apixaban 2.5 mg orally twice daily	813	Incidence of VTE. Incidence of major or clinically relevant nonmajor bleeding. (10–13 days after surgery)	Post-op osocimab 0.3 mg/kg: 23.7% Post-op osocimab 0.6 mg/kg: 15.7% Post-op osocimab 1.2 mg/kg: 16.5% Post-op osocimab 1.8 mg/kg: 17.9% Pre-op osocimab 0.3 mg/kg: 29.9% Pre-op osocimab 1.8 mg/kg: 11.3% Enoxaparin: 26.3% Apixaban: 14.5% (postoperative osocimab 0.6 mg/kg, 1.2 mg/kg, and 1.8 mg/kg met criteria for noninferiority, and the preoperative 1.8-mg/kg dose of osocimab met criteria for superiority compared with enoxaparin)	Post-op 0.3: 2% Post-op 0.6: 0% Post-op 1.2: 1% Post-op 1.8: 3% Pre-op 0.3: 1.9% Pre-op 1.8: 4.7% Enoxaparin: 5.9% Apixaban: 2%
	ANT-005 TKA [28]	Abelacimab 30, 75, 150 mg post-operative IV infusion	Enoxaparin 40 mg S.C once daily	412	Incidence of VTE (8–12 days after surgery). Incidence of major or clinically relevant nonmajor bleeding (up to 30 days after surgery)	Abelacimab 30 mg: 13% Abelacimab 75 mg: 5% Abelacimab 150 mg: 4% Enoxaparin 40 mg: 22% (The 30-mg abelacimab regimen was noninferior, and the 75-mg and 150-mg abelacimab regimens were superior to enoxaparin)	Abelacimab 30 mg: 2% Abelacimab 75 mg: 2% Abelacimab 150 mg: 0% Enoxaparin: 0%
	AXIOMATIC-TKR [57]	Milvexian 25, 50, 100, 200 mg orally twice daily and 25, 50, 200 mg orally once daily	Enoxaparin 40 mg S.C once daily	1242	Incidence of VTE. Incidence of bleeding of any severity. (10–14 days after surgery)	Milvexian 25 mg twice daily: 21% Milvexian 50 mg twice daily: 11% Milvexian 100 mg twice daily: 9% Milvexian 200 mg twice daily: 8% Milvexian 25 mg once daily: 25% Milvexian 50 mg once daily: 24% Milvexian 200 mg once daily: 7% Enoxaparin 40 mg: 21% (The VTE incidence with the twice daily milvexian regimen, was significantly lower than the prespecified benchmark of 30%)	Milvexian: 4% Enoxaparin: 4%
ESRD	Walsh et al. [55]	IONIS-FXIRx 200 and 300 mg S.C	Placebo	43	Pharmacokinetics, pharmacodynamics including reduction levels of FXI activity. Frequency and severity of adverse events, including bleeding events (at 12 weeks)	Mean levels of FXI activity decreased 56.0% (200 mg), 70.7% (300 mg) compared to a 3.9% reduction in the placebo group. (IONIS-FXIRx resulted in dose-dependent prolongation of aPTT and significantly reduced FXI activity compared to placebo)	Clinically relevant bleeding occurred in 0% (200 mg), 6.7% (300 mg), and 7.7% (placebo group)
	Lorentz et al. [23]	Xisomab 3G3 0.25 and 0.5 mg/kg IV	Placebo	24	Number and severity of adverse events, including hemostatic safety (time to achieve hemostasis) at the vascular access site after each hemodialysis session and bleeding events	The incidence of occlusive events requiring circuit exchange decreased from 12.5% to 4% in the 0.25 mg/kg group and from 29% to 12.5% in the 0.5 mg/kg group while with Placebo, the incidence remained the same	No clinically relevant bleeding event was reported in the study group
AF	PACIFIC-AF [30]	Asundexian 20 and 50 mg orally once daily	Apixaban 5 mg twice daily	755	The composite of major or CRNMB according to ISTH criteria (at 12 months)	Asundexian 20 mg: 1.2% Asundexian 50 mg: 0.39% Apixaban: 2.4% (Asundexian at doses of 20 and 50 mg once daily resulted in statistically significant lower rates of bleeding compared with standard dosing of apixaban)	Asundexian resulted in lower rates of bleeding events The rate of any adverse events was similar in the groups (47%, 47% and 49%)
Acute non-cardioembolic ischemic stroke	PACIFIC-Stroke [31]	Asundexian 10, 20 and 50 mg orally once daily	Placebo	1808	The composite of incident MRI-detected covert brain infarcts and recurrent symptomatic ischemic stroke (at or before 26 weeks) Incidence of major or CRNMB according to ISTH criteria	Asundexian 10 mg: 19% Asundexian 20 mg: 22% Asundexian 50 mg: 20% Placebo: 19% (Asundexian did not reduce the composite endpoint of covert brain infarction or ischemic stroke)	Asundexian 10 mg: 4% Asundexian 20 mg: 3% Asundexian 50 mg: 4% Placebo: 2%
Acute ischemic stroke or high-risk TIA	AXIOMATIC-SSP* [33]	Milvexian 25 mg orally once daily and 25, 50, 100, 200 mg twice daily	Placebo	2366	The composite of incident ischemic stroke or new covert brain infarction on MRI (at 3 months) Incidence of major bleeding defined as BARC types 3 or 5 bleeding	Milvexian 25 mg once daily: 16.2% Milvexian 25 mg twice daily: 18.5% Milvexian 50 mg twice daily: 14.1% Milvexian 100 mg twice daily: 14.7% Milvexian 200 mg twice daily: 16.4% Placebo: 16.6% (Milvexian did not significantly reduce the composite outcome of symptomatic ischemic stroke or covert brain infarction)	25 mg once: 0.6% 25 mg twice: 0.6% 50 mg twice: 1.5% 100 mg twice: 1.6% 200 mg twice: 1.5% Placebo: 0.6%
Acute myocardial infarction	PACIFIC-AMI [32]	Asundexian 10, 20 and 50 mg orally once daily	Placebo	1601	The composite of cardiovascular death, MI, stroke, or stent thrombosis Incidence of BARC type 2, 3, or 5 bleeding (At 12 months	Asundexian 10 mg: 6.8% Asundexian 20 mg: 6.0% Asundexian 50 mg: 5.5% Placebo: 5.5% (Asundexian resulted in numerically lower rates of the composite endpoint but, no statistically significant difference was found compared to placebo)	Asundexian 10 mg: 7.6% Asundexian 20 mg: 8.1% Asundexian 50 mg: 10.5% Placebo: 9.0%

*VTE* venous thromboembolism, *CRNMB* clinically relevant non-major bleeding, *ISTH* International Society on Thrombosis and Hemostasis, *BARC* Bleeding Academic Research Consortium.

* The results of AXIOMATIC-SSP were presented at the European Society of Cardiology 2022 Conference: August 28, 2022.

### TKA

Four clinical trials involving four different drugs (IONIS-FXIRx, Osocimab, Abelacimab, and Milvexian) have been published [9, 25, 28, 35].

- **FXI-ASO TKA (NCT01713361):** an open-label, parallel-group noninferiority randomized controlled trial in which 300 patients undergoing elective TKA, were randomly assigned to receive one of three doses of FXI-ASO (100 mg, 200 mg, and 300 mg) or 40 mg of enoxaparin once daily. The primary efficacy endpoint was the incidence of adjudicated total VTE, which was a composite of objectively confirmed symptomatic VTE, unexplained death for which pulmonary embolism could not be ruled out, and asymptomatic DVT (detected by mandatory bilateral venography at 8–12 days postoperatively). Major or clinically relevant nonmajor bleeding (CRNMB) were identified as the primary safety outcomes. Shortly after starting the trial, the 100-mg dose regimen was discontinued to ensure a sufficient reduction in factor XI levels during and after surgery. Treatment with FXI-ASO was initiated 36 days before surgery and patients received a total of 9 subcutaneous doses over a period of 39 days. Meanwhile, Enoxaparin, at a dose of 40 mg administered subcutaneously once daily, was initiated the evening before or 6 to 8 h after surgery and was to be continued for at least 8 days postoperatively. The incidence rates of VTE were quite comparable with 27% and 30% in 200 mg of FXI-ASO and enoxaparin groups, respectively, whereas the incidence was significantly reduced to only 4% in the 300 mg of FXI-ASO group. By contrast, the incidence of bleeding events was 3% in both groups of FXI-ASO compared to 8% in the enoxaparin group. Frequent occurrences of mild injection site reactions were noted among participants using FXI-ASO, but these reactions did not lead to the discontinuation of the study drug. The dose regimen's safety profile was further supported by the similarity of postoperative hemoglobin levels between FXI-ASO and enoxaparin users.
- **FOXTROT (NCT03276143):** Randomized, open-label, adjudicator-blinded, phase 2 noninferiority trial in which 813 adult patients, who were undergoing unilateral knee arthroplasty, were randomly assigned to receive single intravenous osocimab postoperative doses of 0.3, 0.6, 1.2, or 1.8 mg/kg; preoperative doses of 0.3 or 1.8 mg/kg; or 40 mg of subcutaneous enoxaparin once daily or 2.5 mg of oral apixaban twice daily for at least 10 days or until venography. The primary outcome was VTE incidence (detected by mandatory bilateral venography at 10 to 13 days after surgery, confirmed symptomatic DVT, or pulmonary embolism). A noninferiority margin of 5% was selected for comparison with enoxaparin. The main safety outcome evaluated was the occurrence of major or CRNMB within 10 to 13 days after the surgery. The primary outcome occurred in 23.7% receiving 0.3 mg/kg, 15.7% receiving 0.6 mg/kg, 16.5% receiving 1.2 mg/kg, and 17.9% receiving 1.8 mg/kg of osocimab postoperatively; 29.9% receiving 0.3 mg/kg and 11.3% receiving 1.8 mg/kg of osocimab preoperatively; 26.3% receiving enoxaparin; and 14.5% receiving apixaban. Hence, postoperative osocimab 0.6 mg/kg, 1.2 mg/kg, and 1.8 mg/kg were deemed noninferior compared to enoxaparin, and the preoperative 1.8-mg/kg dose of osocimab showed superiority over enoxaparin; however, Postoperative and preoperative doses of 0.3 mg/kg of osocimab did not meet the predetermined criteria for noninferiority. The incidence of major or CRNMB was observed in up to 4.7% of patients receiving osocimab, 5.9% receiving enoxaparin, and 2% receiving apixaban. Bleeding was linked to the surgical site. No intracranial bleeding or bleeding into another critical site was observed.
- **ANT-005 TKA (EudraCT 2019–003756-37):** an open-label, parallel-group noninferiority trial in which 412 patients who were undergoing TKA were randomly assigned to receive either a single intravenous dose of abelacimab (30 mg, 75 mg, or 150 mg) postoperatively or 40 mg of enoxaparin subcutaneously once daily. The primary efficacy outcome was VTE (detected by mandatory bilateral venography at 8 to 12 days after surgery, confirmed symptomatic DVT, or pulmonary embolism), and the primary safety outcome was major or CRNMB up to 30 days after surgery. Incidence of VTE was 13%, 5%, 4%, and 22% for 30, 75, 150 mg of abelacimab, and 40 mg of enoxaparin, respectively. Bleeding occurred in 2%, 2%, and 0% of the patients in the 30, 75, and 150 mg abelacimab groups, respectively, and no bleeding event was reported in the enoxaparin group. Serious adverse events occurred in 1%, 3%, and 1% of the patients in the 30, 75, and 150 mg abelacimab groups, respectively, and in none of the patients in the enoxaparin group. No anti-drug antibodies or hypersensitivity reactions were detected.
- **AXIOMATIC-TKR (NCT03891524):** a parallel-group, phase 2 trial in which 1242 patients, who were undergoing knee arthroplasty, were randomly assigned to receive one of seven postoperative regimens of milvexian (25, 50, 100, or 200 mg twice daily or 25, 50, or 200 mg once daily) or enoxaparin (40 mg once daily). The primary efficacy outcome was VTE. The primary safety outcome was bleeding events whereas the primary efficacy endpoint was VTE incidence, which was a composite of asymptomatic DVT (detected by mandatory unilateral venography performed at 10 to 14 days postoperatively), confirmed symptomatic DVT, or death from any cause. The proof of efficacy was determined as a significant dose–response relationship and a venous thromboembolism incidence that was significantly lower than 30% with the twice-daily milvexian regimen. In the milvexian twice-daily group, venous thromboembolism developed in 21%, 11%, 9%, and 8% among patients received 25, 50, 100, and 200 mg respectively. On the other hand, in the milvexian once daily group, venous thromboembolism developed in 25%, 24%, and 7% among patients received 25, 50, and 200 mg respectively, as compared to 21% in patients taking enoxaparin. Bleeding of any severity was reported in 4% of patients taking milvexian; the same incidence with enoxaparin. Major or clinically relevant nonmajor bleeding was reported in 1% and 2%, respectively; and serious adverse events were reported in 2% and 4%, respectively. Milvexian increased the aPTT ratio in a dose-dependent manner, and no evidence of a dose-dependent increase in bleeding was noted.

It’s worth mentioning that the trials in patients undergoing TKA predominantly identified VTE events through screening venography 8-14 days after surgery, mostly asymptomatic and non-clinically significant which is an important limitation in these phase 2 clinical trials. Therefore, emphasis is placed on ongoing clinical trials targeting clinical endpoints in this patient group.

### ESRD

Two clinical trials involving two different drugs IONIS-FXIRx) and Xisomab 3G3) have been published do date [23, 55].

- **Walsh et al. (NCT02553889):** a phase 2b study enrolled 49 patients receiving hemodialysis. The study was composed of two parts. First, 6 individuals received 1 dose of IONIS-FXIRx 300 mg pre- and post-hemodialysis. Subsequently, 43 participants were allocated in a double-blind, randomized design to either 200 mg, 300 mg of IONIS-FXIRx, or placebo for 12 weeks. The principal goal was to evaluate the pharmacokinetics, pharmacodynamics, and adverse events. The PK of the drug was consistent with previous studies and similar whether injected pre- or post-hemodialysis. No accumulation of IONIS-FXIRx was observed after repeated administration. After 12 weeks, mean levels of FXI activity decreased by 56.0% and 70.7% in the 200 mg and 300 mg groups, respectively compared to a 3.9% reduction in the placebo group. Injection site reaction was the most common adverse event. Clinically relevant bleeding occurred in 0 (0.0%; 200 mg), 1 (6.7%; 300 mg), and 1 (7.7%; placebo) patients, respectively.
- (RxFXI-formerly IONIS) The phase 2b study of fesomersen (formerly IONIS-FXIRx)achieved its primary outcome measure of no increase in incidence of major bleeding and clinically relevant non-major bleeding as compared to placebo. Data from the study show that fesomersen, administered monthly at 40 mg, 80 mg, and 120 mg for up to 48 weeks, was safe and well-tolerated. Fesomersen also demonstrated substantial and statistically significant reductions in Factor XI activity levels.
- **Lorentz et al. (NCT03612856):** a randomized, double-blinded, phase 2 trial comparing Xisomab (AB023) to placebo in 24 patients with ESRD undergoing heparin-free hemodialysis. Patients were randomly allocated to receive a single dose of AB023 (0.25 or 0.5 mg/kg) or placebo before dialysis. The primary aim was to evaluate the safety of the drug about bleeding events. No clinically relevant bleeding event was reported in the study group, and the time to hemostasis at vascular access sites remains the same after drug administration. Furthermore, after the drug administration, there were reductions in levels of thrombin-antithrombin complexes and C-reactive protein, and there were fewer occlusive events that necessitated hemodialysis circuit exchange. So, AB023 effectively inhibited contact activation-induced coagulation, was well-tolerated, and resulted in decreased clotting within the dialyzer.

### AF

One clinical trial was published so far investigating the use of asundexian in patients with AF [30].

- **PACIFIC-AF (NCT04218266):** a randomized, double-blind, phase 2 study in which asundexian 20 mg or 50 mg once daily compared to apixaban 5 mg twice daily in 755 patients aged 45 years or older with atrial fibrillation, a CHA2DS2-VASc score of at least 2 if male or at least 3 if female (moderate to high risk for stroke), and increased bleeding risk. Patients were followed-up for 12 weeks. The primary endpoint was the composite of major or clinically relevant non-major bleeding according to the International Society on Thrombosis and Hemostasis (ISTH). The incidence of bleeding events was 1.2% and 0.4% in the asundexian 20 mg and 50 mg respectively compared to 2.4% in the apixaban group which represented a significant reduction in bleeding rates with the 50 mg asundexian regimen. On the other hand, asundexian at a 20 mg dose exhibited 81% inhibition of FXIa activity during trough concentrations and 90% inhibition during peak concentrations, whereas the 50 mg dose showed 92% inhibition during trough concentrations and 94% inhibition during peak concentrations. Regarding the exploratory thrombotic composite endpoint of cardiovascular death, myocardial infarction, ischemic stroke, or systemic embolism, a total of 2, 4, and 3 events occurred in asundexian 20 mg, asundexian 50 mg, and apixaban groups respectively. The small number of events in phase 2 did not raise an alarm, however, the IDMC recommended stopping the OCEANIC AF phase 3 trial due to inferior efficacy on November 19, 2023. The other trials were not impacted and are ongoing (bayer.com; accessed November 20, 2023).

### Acute non-cardioembolic ischemic stroke

One clinical trial was published so far investigating the use of asundexian in patients with acute non-cardioembolic ischemic stroke [31].

- **PACIFIC-Stroke (NCT04304508):** a randomized, double-blind, phase 2b study in which 1808 patients aged 45 years or older with non-cardioembolic ischemic stroke were randomly assigned to receive asundexian 10, 20, 50 mg once daily dose or placebo in addition to usual antiplatelet therapy. Patients were treated and followed up for 26-52 weeks. The composite of recurrent symptomatic ischemic stroke and MRI-detected brain infarcts after randomization was defined as a primary efficacy outcome. The primary safety outcome was major or CRNMB according to ISTH criteria. The incidence of bleeding events was observed in 4%, 3%, and 4% in the asundexian 10, 20, and 50 mg respectively compared to 2% in the placebo group. On the other hand, the primary efficacy outcome was observed in 19%, 22%, and 20% in the asundexian 10, 20, and 50 mg respectively compared to 19% in the placebo group. There was no dose-response association and no significant increase in the incidence of the primary safety outcome.

### Acute ischemic stroke or high-risk TIA

One clinical trial was published so far investigating the use of milvexian in patients with acute ischemic stroke or high-risk TIA [38] (The results were presented at the European Society of Cardiology 2022 Conference; August 28, 2022).

- **AXIOMATIC-SSP (NCT03766581):** a phase 2, randomized, double-blind, placebo-controlled trial examining the efficacy and safety of milvexian in 2366 patients with acute ischemic stroke or at high risk of TIA. The main objective was to estimate the dose-response relationship of milvexian on stroke occurrence and bleeding. Patients were randomly assigned to receive one of five doses of milvexian (25, 50, 100, 200 mg twice daily, or 25 mg once daily) or placebo daily for 90 days. All study participants received background treatment with aspirin and clopidogrel for 21 days, followed by aspirin from day 22 to 90. The primary efficacy outcome was the composite of ischemic stroke or incident infarct on brain MRI. The primary safety outcome was the major bleeding events defined as Bleeding Academic Research Consortium (BARC) type 3 or 5 bleeding. The primary efficacy outcome was lower at the 50 and 100 mg twice daily groups; however, there was no apparent dose-response relationship (placebo 16.6%, 25 mg once daily 16.2%, 25 mg twice daily 18.5%, 50 mg twice daily 14.1%, 100 mg twice daily 14.7%, 200 mg twice daily 16.4%). Additionally, milvexian numerically reduced the risk of clinical ischemic stroke in the intention-to-treat population at all doses except 200 mg twice daily, with doses from 25 to 100 mg twice daily showing an approximately 30% relative risk reduction compared to placebo (placebo 5.5%, 25 mg once daily 4.6%, 25 mg twice daily 3.8%, 50 mg twice daily 4.0%, 100 mg twice daily 3.5%, 200 mg twice daily 7.7%). On the other hand, the incidence of major bleeding was low overall (placebo 0.6%, 25 mg once daily 0.6%, 25 mg twice daily 0.6%, 50 mg twice daily 1.5%, 100 mg twice daily 1.6%, 200 mg twice daily 1.5%). The incidence of major bleeding for the milvexian 25 mg once and twice daily groups was like placebo, while a moderate increase in the 50 mg twice daily group and above with no dose-response relationship. There was no increase in severe bleeding (BARC type 3c or symptomatic intracranial hemorrhage) compared to placebo, and there was no fatal bleeding in any arm of the study.

### Acute myocardial infarction

One clinical trial was published so far investigating the use of asundexian in patients with acute myocardial infarction [32].

- **PACIFIC-AMI (NCT04304534):** a double-blind, placebo-controlled, phase 2, dose-ranging trial investigating the efficacy and safety of asundexian in 1601 patients with recent acute myocardial infarction. The primary efficacy outcome was a composite of cardiovascular death, MI, stroke, or stent thrombosis. The main safety outcome was BARC type 2, 3, or 5 bleeding. Patients were randomly assigned to receive oral asundexian 10, 20, or 50 mg or placebo once daily for 6 to 12 months. asundexian was associated with a dose-dependent reduction of FXIa activity with 50 mg of asundexian resulted in > 90% inhibition of baseline FXIa activity. There was no significant difference in the incidence of bleeding among different groups with incidence of 7.6%, 8.1%, 10.5%, and 9.0% in patients receiving asundexian 10 mg, 20 mg, 50 mg, and placebo respectively (pooled asundexian versus placebo: hazard ratio, 0.98 [90% CI, 0.71-1.35]). Whereas the efficacy outcome identified in 6.8%, 6.0%, 5.5%, and 5.5% in patients receiving asundexian 10 mg, 20 mg, 50 mg, and placebo respectively (pooled asundexian versus placebo: hazard ratio, 1.05 [90% CI, 0.69-1.63). Therefore, asundexian was associated with a dose-dependent, near-complete inhibition of FXIa activity without a significant increase in bleeding and a low rate of ischemic events.

### Ongoing randomized controlled trials

Several randomized controlled trials are currently underway investigating FXI inhibitors’ efficacy and safety for VTE prophylaxis in different clinical conditions. These conditions include ESRD, ischemic stroke, AF, and cancer. A summary of these trials including drug name, control, number of participants, clinical scenario, outcome measures, follow-up period, and trials timelines is shown in (Table 3).

**Table 3:** Ongoing randomized controlled trials on factor XI inhibitors

Class	Drug	Trial	Indication	N	Comparator	Primary outcome and safety measures	Follow-up	Proposed or actual completion date
Antisense oligonucleotides (ASO)	IONIS-FXIRx	NCT03358030 (Phase 2)	ESRD	213	placebo	Major and CRNM bleeding, treatment-emergent adverse events	260 days	January 2023*
	Fesomersen	NCT04534114 (Phase 2b)	ESRD	307	placebo	Composite of Major Bleeding and CRNM Bleeding	48 weeks	July 2023*
Small molecule oral drugs	Milvexian	NCT05702034 (Phase 3)	Acute ischemic stroke or high-risk TIA	15,000	placebo	Time to First Occurrence of ischemic stroke	41 months	December 2026
	Asundexian	NCT05686070 (Phase 3)	Acute Non-cardioembolic Ischemic Stroke or High-risk TIA	9300	Placebo	Time to first occurrence of ischemic stroke, time to first occurrence of ISTH major bleeding	31 months	October 2025
		NCT05643573 (Phase 3)	AF	18,000	Apixaban	Time to first occurrence of composite of stroke or systemic embolism, time to first occurrence of ISTH major bleeding	34 months	January 2024* (Stopped early due to lack of efficacy)
	SHR2285	NCT05203705 (Phase 2)	TKA	500	Enoxaparin	Composite of DVT, VTE, and non-fatal PE Composite of major and CRNM bleeding	42 days	May 2023
Monoclonal antibodies	Osocimab	NCT04523220 (Phase 2)	ESRD	704	placebo	Composite of Major Bleeding and CRNM Bleeding, Composite of moderate and severe adverse events	6 months	May 2022*
	Abelacimab	NCT04755283 (Phase 2)	AF	1200	Rivaroxaban	Rate of major or CRNM bleeding events	17 months	January 2025
		NCT05712200 (Phase 3)	AF	1900	Placebo	Time to first event of ischemic stroke or systemic embolism, time to first occurrence of BARC type 3c/5 bleeding	30 months	March 2025
		NCT05171049 (Phase 3)	cancer associated VTE	1655	Apixaban	Time to first event of centrally adjudicated VTE recurrence	6 months	September 2025
		NCT05171075 (Phase 3)	cancer associated VTE	1020	Dalteparin	Time to first event of centrally adjudicated VTE recurrence	6 months	September 2025
	MK-2060	NCT05027074 (Phase 2)	ESRD	489	placebo	Time to First AVG Thrombosis	34 months	October 2024
	REGN9933	NCT05618808 (Phase 2)	TKA	360	Enoxaparin/ Apixaban	Incidence confirmed, adjudicated of VTE Incidence of major or CRNM bleeding	75 days	August 2024

*ESRD* end-stage renal disease, *TKA* total knee arthroplasty, *AF* atrial fibrillation, *TIA* transient ischemic attack, *VTE* venous thromboembolism, *DVT* deep vein thrombosis, *PE* pulmonary embolism, *CRNM* clinically relevant Non-Major, *ISTH* International Society on Thrombosis and Hemostasis, *BARC* Bleeding Academic Research Consortium, *AVG* arteriovenous graft.

*Indicates the actual study completion date.

## Conclusion

Despite the availability of various oral and parenteral anticoagulants for preventing venous and arterial thromboembolism, bleeding complications continue to pose challenges for both healthcare providers and patients. A novel approach in anticoagulation involves targeting the contact pathway at the level of FXI to uncouple hemostasis and thrombosis has emerged as a potential safer option. Promising results from the completed phase 2 studies of FXI inhibitors, including antisense oligonucleotides, monoclonal antibodies, and small molecules, indicate dose-dependent reductions in FXI concentrations and thrombotic complications without a corresponding increase in the bleeding risk. The ongoing phase 2 and 3 trials focusing on clinical endpoints will be crucial in defining the role of FXI inhibitors in the future and clearly delineating both their overall efficacy and safety profiles. These developments may potentially pave the way for safer and more effective anticoagulant therapies for many patients in the future. Moreover, the development of reversal agents especially for drugs with long half-lives is another important issue that should be investigated.

### Future directions

The biological and pathophysiological foundation for FXI inhibitors and their development for clinical use is very strong. The initial focus for clinical indications is well-founded based on opportunities and unmet need-secondary prevention of ischemic stroke, atrial fibrillation, cancer-associated thrombosis, and both primary and secondary prevention in patients with ESRD. Each may be met with enthusiasm by researchers, clinicians, and patients alike. The ability of FXI inhibitors to replace DOACs in atrial fibrillation for example will require at the very least similar efficacy and superior safety in patients at moderate (or high) risk for both cardioembolic stroke and major bleeding. The use of FXI inhibitors in patients with ESRD, and hopefully those with moderately reduced kidney performance in the future, is particularly exciting given the high risk for thrombotic events at several anatomic sites and concomitant bleeding risk in these patients.

## Data Availability

The authors confirm that the data supporting the findings of these studies previously published are available within the article [and/or] its supplementary materials.
